# The evolving hematopoietic niche during development

**DOI:** 10.3389/fmolb.2024.1488199

**Published:** 2024-10-02

**Authors:** Raúl Sánchez-Lanzas, Amanda Jiménez-Pompa, Miguel Ganuza

**Affiliations:** Centre for Haemato-Oncology, Barts Cancer Institute, Queen Mary University of London, London, United Kingdom

**Keywords:** hematopoietic stem cells, developmental hematopoiesis, hematopoietic niches, aorta-gonad-mesonephros, fetal liver, bone marrow

## Abstract

Mammalian hematopoietic stem cells (HSCs) emerge from the hemogenic endothelium in the major embryonic arteries. HSCs undergo a complex journey first migrating to the fetal liver (FL) and from there to the fetal bone marrow (FBM), where they mostly remain during adult life. In this process, a pool of adult HSCs is produced, which sustains lifelong hematopoiesis. Multiple cellular components support HSC maturation and expansion and modulate their response to environmental and developmental cues. While the adult HSC niche has been extensively studied over the last two decades, the niches present in the major embryonic arteries, FL, FBM and perinatal bone marrow (BM) are poorly described. Recent investigations highlight important differences among FL, FBM and adult BM niches and emphasize the important role that inflammation, microbiota and hormonal factors play regulating HSCs and their niches. We provide a review on our current understanding of these important cellular microenvironments across ontogeny. We mainly focused on mice, as the most widely used research model, and, when possible, include relevant insights from other vertebrates including birds, zebrafish, and human. Developing a comprehensive picture on these processes is critical to understand the earliest origins of childhood leukemia and to achieve multiple goals in regenerative medicine, such as mimicking HSC development *in vitro* to produce HSCs for broad transplantation purposes in leukemia, following chemotherapy, bone marrow failure, and in HSC-based gene therapy.

## Introduction

**FIGURE 1:**
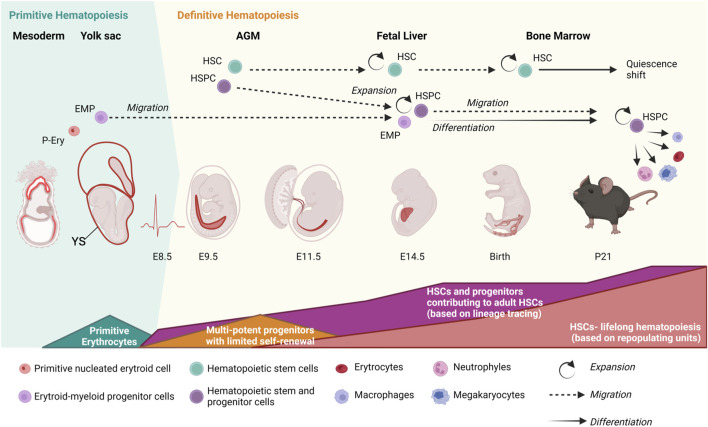
Developmental waves of hematopoiesis. Primitive erythroid cells (P-Ery) and erythroid-myeloid progenitors (EMPs) with limited contribution to adult hematopoiesis emerge in the yolk sac (YS). The presence of hematopoietic stem cells (HSCs), characterized by their ability to repopulate lethally irradiated recipients, is first detected around embryonic day of development (E) 10.5 in the embryo once circulation is stablished (∼E8.5). HSCs emerge from the hemogenic endothelium in the major arteries of the embryo (including aorta, located in the aorta-gonad-mesonephros region, AGM, and umbilical and vitelline arteries). Lineage-tracing studies detected a much higher number of HSC precursors in the embryo than assumed by classical estimates based on repopulating units. Shapes at the bottom indicate the duration and magnitude of each developmental wave. HSCs later migrate to the fetal liver (starting around E12.5), where they mature and modestly expand in numbers according to lineage tracing studies. From the fetal liver, HSCs start to migrate to the bone marrow around E15.5, where they will mostly remain during adulthood. Around 3–4 weeks post- birth, HSCs undergo a quiescence shift to preserve their cellular and molecular integrity and population size. Most adult HSCs are quiescent and only abandon this state to reinstate homeostasis in a controlled manner following insults (e.g., bleeding, inflammation, infections, cellular turnover). Embryonic days of development refer to mouse ontogeny. Processes seem preserved in other mammals. Figure created with Biorender.

In mammals at least three embryonic developmental waves of hematopoiesis produce hematopoietic cells with different contribution in the life of an organism. The first two waves emerge in the yolk sac (YS) produce primitive nucleated erythroid cells and erythroid-myeloid progenitor cells (EMPs) (Medvinsky et al., 2011) (Figure 1). Most YS-derived hematopoietic cells will serve to cope with embryonic needs, including oxygen distribution in growing embryos, and will have little contribution to adulthood. Yet, some YS-derived cells such as tissue-resident macrophages, with self-renewing activity, will persist during adult life (Medvinsky et al., 2011). Hematopoietic stem cells (HSCs), which sustain life-long hematopoiesis, emerge in a third wave of hematopoiesis (known as definitive hematopoiesis) (Medvinsky et al., 2011) when a fraction of endothelial cells (the hemogenic endothelium) located in the major arteries of the embryo, including the aorta and vitelline and umbilical arteries in mammals, undergo endothelial-to-hemogenic transition (EHT) (Ganuza et al., 2020; Ottersbach, 2019; Simic et al., 2020). Newly formed HSCs and hematopoietic stem and progenitor cells (HSPCs) migrate via blood circulation to the fetal liver (FL) where they mature and expand in number, although recent breakthroughs indicate that this expansion is modest (only ∼2 fold) and HSCs divide asymmetrically (Ganuza et al., 2020; Ganuza et al., 2022a; Patel et al., 2022; Ganuza et al., 2022b) (Figure 1). Later in gestation, HSCs relocate to the bone marrow (BM) where, under healthy conditions, remain during adult life (Figure 1). In the BM, the HSC pool expands in number before undergoing a “quiescence shift” that HSCs will only abandon to maintain or reinstate the homeostasis of the hematopoietic system following acute insults (e.g., bleeding, bacterial and viral infections…) or more stable and continuous damages such as cell turnover and chronic inflammation (Ganuza et al., 2022b; Bowie et al., 2006; Calvanese et al., 2014; Johnson et al., 2020; Fernandez Sanchez et al., 2024; Bogeska et al., 2022; Ganuza and McKinney-Freeman, 2017).

Over these various stages of development, HSCs and their progenitors receive critical cues including cytokines, chemokines, growth factors and ligand-receptor interactions that first trigger their specification and later regulate their proliferative state and behavior (e.g., quiescence versus cell division and differentiation) (Clements and Khoury, 2024; Mikkola and Orkin, 2006; Calvanese and Mikkola, 2023; Agrawal et al., 2024). The cellular microenvironment where HSCs locate and that provide specific supporting signals constitutes the HSC-niche. Thus, the HSC-niche integrates cues received from the organism to maintain the homeostasis of the hematopoietic system. The HSC-niche dramatically changes during development, especially considering the journey of HSCs across multiple embryonic locations (Mikkola and Orkin, 2006). Although the adult HSC-niche has been widely studied and numerous reviews are available on the topic (Sanchez-Lanzas et al., 2022; Morrison and Scadden, 2014; Pinho and Frenette, 2019; Lucas, 2017), HSC-niches including those related to the specification of HSCs in the major embryonic arteries (Clements and Khoury, 2024; Jaffredo et al., 2013) and the FL-(Agrawal et al., 2024), fetal BM (FBM)-, perinatal- and infant-BM are much less characterized (Gao et al., 2018). In this Review Article we provide a comprehensive overview on the current knowledge of the various HSC-niches during ontogeny, with a focus on pre-adult HSC niches. We describe the major biological and developmental events taking place at each stage and the cellular and molecular niche components that constitute those microenvironments and how they are believed toshape HSC behavior. We focus mostly on the mouse as the best characterized mammalian system and provide additional insights on humans and other non-mammalian vertebrates as zebrafish and birds when possible. The reader can find key terms described in Table 1.

**TABLE 1:** Glossary.

Key terms and concepts	Definition/background
AGM (Aorta-gonad-mesonephros)	Anatomical structure in the embryo comprised by the dorsal aorta and urogenital ridges and kidney rudiments Medvinsky et al. (2011)
Dorsal aorta	Embryonic artery located in the AGM. It harbors hemogenic endothelium from which HSC arise Medvinsky et al. (2011)
EHT (endothelial to hematopoietic transition)	Process though which a fraction of the hemogenic endothelium acquires hematopoietic properties as detected by the ability of the newly differentiated progenitors to produce hematopoietic cells Medvinsky et al. (2011)
HSCs (hematopoietic stem cells)	Multipotent cells with the ability to repopulate the whole hematopoietic system of a lethally irradiated recipient mouse. This includes the production of cells of the three blood lineages: myeloid, B-cells and T-cells Medvinsky et al. (2011)
Transplantation of embryonic tissues	Dissociated embryonic tissues are transplanted into lethally irradiated recipients to assess the presence and ability of the transplanted tissues to repopulate the hematopoietic system of a recipient mouse Ganuza et al. (2020); Ganuza et al. (2022b)
RUs (repopulating units)	Number of transplanted cells with hematopoietic stem cell activity. To estimate the number of repopulating units, several cohorts of mice are transplanted with different numbers of cells in a classic limiting dilution fashion. Based on poisson statistics this allows to estimate the frequency of stem cells among the mix of transplanted cells. Multicolor-based lineage tracing (Confetti) also allows to obtain similar estimates Ganuza et al. (2020); Ganuza et al. (2022b); Ganuza et al. (2017)
Fate versus potential	The detection of RUs only informs on the potential of the transplanted cells to perform as HSC at the moment of transplantation. Classical transplantation assays do not provide information on the fate of the embryonic HSCs and cannot predict if they will contribute or not to adult hematopoiesis. Classic transplantation also fails to detect the presence of immature cells (not mature enough to repopulate the hematopoietic system of a recipient at the moment of transplantation) that if they were to stay in the embryo they would have contributed to the adult HSC pool Ganuza et al. (2020); Ganuza et al. (2022b); Ganuza et al. (2017)
Lineage-tracing technologies	This includes the use of genetically engineered reporter mice and tracing of natural barcodes based on the natural acquisition of mutations and of lentivirally inserted barcodes. Lineage-tracing technologies can inform on the fate of the labeled cells and on their functional output including their contribution to differentiated lineages Ganuza et al. (2020); Ganuza et al. (2022b); Sanchez-Lanzas et al. (2022)
Fetal liver (FL)	HSCs and non-mature HSC precursors migrate from the AGM into the FL where HSCs and precursors mature and according to recent reports modestly expand in number Ganuza et al. (2022a); Lee-Six et al. (2018)
Caudal hematopoietic tissue	Organ equivalent to the mammalian FL and where HSCs expand Medvinsky et al. (2011); Clements and Khoury (2024)
Fetal bone marrow (FBM)	The bone marrow is the final destination of the HSCs during their developmental journey and where they will mainly reside during adult life. Major differences have been detected between the FBM and the adult bone marrow (BM) Hall et al. (2022)
Whole kidney marrow	Equivalent to mammalian BM and the site for hematopoiesis in the adult zebrafish Medvinsky et al. (2011); Clements and Khoury (2024)
Quiescence shift	HSCs experience a transition into a quiescent stage around 3–4 weeks post-birth in mice and post-adolescence in human. This transition has been interpreted to maintain the genomic integrity of the HSC pool Bowie et al. (2006); Bowie et al. (2007)

### The HSC-specification niche: aorta-gonad-mesonephros

**FIGURE 2:**
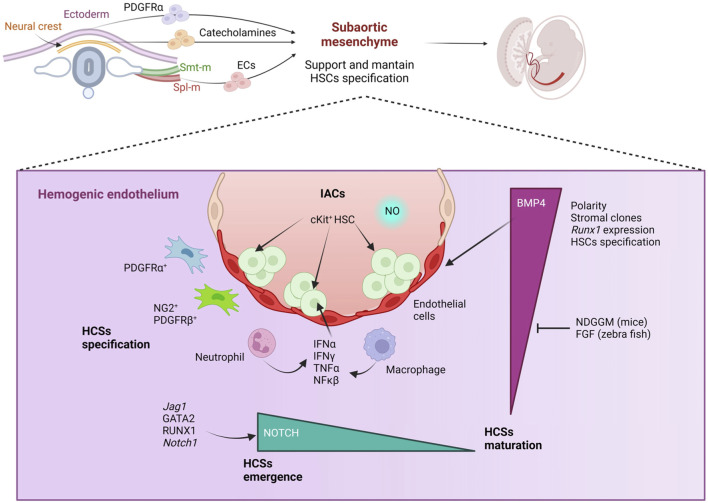
The hematopoietic stem cell (HSC) niche during HSC specification. The subaortic mesenchyme derived from the splachnopleural mesoderm, plays a key role in the establishment of a dorsoventral polarity in the aorta, which eventually leads to the emergence of HSCS from the hemogenic endothelium, located in the ventral aspect of the dorsal aorta, through a process known as endothelial-to-hematopoietic transition (EHT). This can be visualized as intra-aortic clusters (IACS) of c-Kit^+^ cells budding into the lumen of the aorta in midgestation mammalian embryos. Migratory ectoderm-derived neural crest cells guided to the ventral aspect of the aorta by platelet-derived growth factor receptor a (PDGFRα) are also required during EHT. Catecholamines produced by neural crest-derived neurons, supports HSCs maintenance post-specification. Bone morphogenic protein-4 (BMP4), GATA2 and NOTCH signaling play a key role in EHT and modulate RUNX1 expression. Both BMP and NOTCH activity need to be downregulated to allow HSC maturation following specification. Sterile inflammation, mediated by various cytokines produced by endothelial and myeloid cells (such as macrophages) also promotes HSC specification. Blood circulation triggering shear stress activating nitric oxide (NO) pathway in the hemogenic endothelium is also critical in EHT. Figure created with Biorender.

HSCs are mesodermal in origin and derive from a subset of endothelial cells (ECs) known as the hemogenic endothelium (HE) in the major embryonic arteries, including vitelline and umbilical arteries and the aorta (Medvinsky et al., 2011). The specific requirement of a supportive niche in HSC specification has been most directly demonstrated in avian embryos by the use of quail-chick chimeras (Pardanaud and Dieterlen-Lievre, 1999), in mice via *in vitro* explant-reaggregates cultures (Matsuoka et al., 2001; Peeters et al., 2009; Souilhol et al., 2016a; Taoudi and Medvinsky, 2007; Ivanovs et al., 2014) and in *Xenopus* via transplantation (Turpen et al., 1997). Table 2 summarizes the methods that have been employed to identify hematopoietic niches during development. We also refer the reader to our previous review article focused on classic and novel approaches to study the bone marrow niche (Sanchez-Lanzas et al., 2022). HSC specification mainly concentrates to the ventral aspect of the dorsal aorta (Jaffredo et al., 2013) (Figure 2). The establishment of a dorsoventral polarity in the dorsal aorta is key in HE commitment (Yvernogeau et al., 2023). It has been shown that the subaortic mesenchyme contributes to this process and during HSC specification (Jaffredo et al., 2013; Ivanovs et al., 2014; Chandrakanthan et al., 2022; Damm and Clements, 2017; Gonzalez Galofre et al., 2024; Kapeni et al., 2022; Miladinovic et al., 2024; Richard et al., 2013; Sa da Bandeira et al., 2022; Crosse et al., 2023; Crosse et al., 2020). The subaortic mesenchyme derives from the splanchnopleural mesoderm, as shown by studies on avian embryos, and contains mesenchymal cells harboring various differentiation potentials including the ability to generate osteoblasts, adipocytes and ECs (Jaffredo et al., 2013; Richard et al., 2013; Mendes et al., 2005; Durand et al., 2006; Pardanaud et al., 1996; Jaffredo et al., 2010). Cells with other embryonic origin also migrate to the subaortic mesenchyme and have a role on HSC specification (Figure 2). Particularly, migratory ectoderm-derived neural crest cells associate with the ventral aspect of the dorsal aorta before HSC specification in zebrafish embryos (Damm and Clements, 2017). Their migration requires platelet-derived growth factor receptor α (PDGFRα) signaling and blocking this migration leads to lack of HSC specification (Damm and Clements, 2017). Additionally, catecholamines produced by the sympathetic nervous system neurons (derived from a fraction neural crest cells) are required to maintain HSCs in the mouse embryo after HSC specification (Damm and Clements, 2017; Kapeni et al., 2022; Fitch et al., 2012). While in chick embryos somite-derived ECs migrate to the aorta to replace HE cells post-EHT (Pouget et al., 2006), in zebrafish dermomyotome-derived ECs were recently shown to travel to the dorsal aorta to support EHT from neighboring HE cells. It would be interesting to determine the timing and relevance of this migration in mammalian embryos (Sahai-Hernandez et al., 2023).

**TABLE 2:** Summary of methods used to study the evolving hematopoietic niche.

Method	Analyzed stage	Organism	Strengths	Limitations	References
Quail-chick chimeras	HSC specification	Quail- chick	Functional information on the requirement of anatomic tissues. It allows tracking tissue origin	Limited cellular and molecular resolution	Pardanaud and Dieterlen-Lievre (1999); Pardanaud et al. (1996)
*In vitro* explant-reaggregates cultures derived from mouse embryos	HSC specification	Mouse	Functional information on the requirement of specific cell types. It allows transplantation of emerging cells following *in vitro* culture to assess HSC presence	HSC specification *in vitro* may not fully recapitulate *in vivo* development	Matsuoka et al. (2001); Peeters et al. (2009); Souilhol et al. (2016a); Taoudi and Medvinsky (2007); Ivanovs et al. (2014); Taoudi et al. (2008); McGarvey et al. (2017)
Gene knockdown via morpholinos (antisense oligonucleotides)	HSC specification	Zebrafish	Functional and molecular information. Large scale studies and screens are possible	Off-target effects of morpholinos require validation. Lack of cellular specificity. Injected zebrafish embryos are “equivalent” to germline knockouts	Damm and Clements (2017); Sahai-Hernandez et al. (2023); Lim et al. (2017); Clements et al. (2011); Bertrand et al. (2010)
Genetic deletion of supportive niche factors	HSC specification, FL, FBM, perinatal BM, adult BM, CHT, kidney marrow	Mouse and zebrafish	Molecular and functional information at cellular level	Low-throughput studies. Promoters may be promiscuous, and deletion of factors triggered non-specifically in various cell types. Inducible models are possible and advisable when possible. Lack of spatial information	Souilhol et al. (2016a); Richard et al. (2013); Robert-Moreno et al. (2008); Nakagawa et al. (2006); Souilhol et al. (2016b); Porcheri et al. (2020); Lizama et al. (2015); Ditadi et al. (2015); Kobayashi et al. (2014); Gama-Norton et al. (2015); Sacilotto et al. (2013); Thambyrajah and Bigas (2022); Hou et al. (2020); Espin-Palazon et al. (2014); Li et al. (2014); Mariani et al. (2019); Ulloa et al. (2021); Xue et al. (2017); Blaser et al. (2017); Lee et al. (2021); Neo et al. (2018); Biswas et al. (2020); Ding et al. (2012); Greenbaum et al. (2013); Ding and Morrison (2013); Asada et al. (2017)
Depletion of candidate niche cells via genetic manipulation	HSC specification, FL, FBM, perinatal BM, adult BM, CHT, kidney marrow	Mouse and zebrafish	Functional information	Promoters are normally not specific. Global depletion of some cellular components may activate confounding compensatory mechanisms	Lucas, (2017); Ulloa et al. (2021); Sugiyama et al. (2013); Calvi et al. (2003); Zhang et al. (2003); Mendez-Ferrer et al. (2010); Winkler et al. (2010a); Chen et al. (1998); Visnjic et al. (2004); Saito et al. (2001); Omatsu et al. (2010); Buch et al. (2005); Bruns et al. (2014); Nakamura-Ishizu et al. (2014); Zhao et al. (2014); Fujioka et al. (2011); Calaminus et al. (2012); Asada et al. (2013)
Histological studies	HSC specification, FL, FBM, perinatal BM, adult BM	Human and mouse	Tissue organization. Spatial information	Limited information to identify cell types. Very reduced molecular information	Agrawal et al. (2024); Fomin et al. (2017); Elvevold et al. (2008); Orlic et al. (1982); Kamps and Cooper (1982); Khan et al. (2016)
Two-dimensional imaging	HSC specification, FL, FBM, perinatal BM, adult BM, CHT, kidney marrow	Human, mouse and zebrafish	Stromal and HSC markers and the use of genetic reporter strains allows to determine spatial location and proximity	No functional information. It relies on a limited number of markers to identify cell types and biased for those selected	Gao et al. (2018); Clements et al. (2011); Ulloa et al. (2021); Khan et al. (2016); Iwasaki et al. (2010); Lee et al. (2021); Liu et al. (2022); Zhou et al. (2014); Mizoguchi et al. (2014); Pineault et al. (2019); Shu et al. (2021); Matsushita et al. (2022); Christodoulou et al. (2020); Kara et al. (2023); Zovein et al. (2008); Yokomizo et al. (2011)
Time-lapse imaging	HSC specification, CHT, kidney marrow, BM	Zebrafish, mouse explants and mouse calvarium	Spatial and longitudinal information to reveal cell behavior	Limited molecular information. Cell identification based on a reduced number of markers based normally on genetic fluorescent labelling	Damm and Clements (2017); Xue et al. (2017); Tamplin et al. (2015); Ding et al. (2012); Sugiyama et al. (2006); Lo et al. (2009); Cordeiro Gomes et al. (2016); Christodoulou et al. (2020); Ding and Morrison (2013); Acar et al. (2015); Kunisaki et al. (2013); Duarte et al. (2018); Mendez-Ferrer et al. (2010); Boisset et al. (2010); Kokkaliaris et al. (2020); Xie et al. (2009); Kohler et al. (2009); Winkler et al. (2010b); Chen et al. (2016); Pinho et al. (2018); Upadhaya et al. (2020)
Derivation of stromal cell lines from niche tissues and co-culture with HSCs and HSC progenitors	HSC specification, FL, FBM, perinatal BM, adult BM	Human and mouse	Molecular and functional information	Fails to provide information on actual cell-contact or proximity. *In vitro* cultures may not reproduce *in vivo* conditions	Gao et al. (2018); Moore et al. (1997a); Chagraoui et al. (2003); Moore et al. (1997b); Wineman et al. (1996); Nolta et al. (2002); Hackney et al. (2002); Chou and Lodish (2010); Chou et al. (2013); Zhang and Lodish (2004); Sugiyama et al. (2011); Agrawal et al. (2024); Fantin et al. (2021); Iwasaki et al. (2010); Choi et al. (2021); Zhang et al. (2006)
Transplantation of stromal components	Adult BM	Mouse	Molecular and functional information	Lacks spatial information	Mendez-Ferrer et al. (2010); Friedenstein et al. (1968); Friedenstein et al. (1974); Tavassoli and Crosby (1968); Maniatis et al. (1971); El-Badri et al. (1998); Sacchetti et al. (2007); Chan et al. (2009); Pinho et al. (2013); Wolf (1978)
Single-cell sequencing coupled with spatially resolved transcriptomics	HSC specification, FL, FBM, perinatal BM, adult BM, CHT, kidney marrow	Human, mouse and zebrafish	Molecular and functional information at single-cell level	Lack of spatial information. Cellular interactions are based on predictions. Rare populations are normally not captured in bulk samples due insufficient sequencing depth	Agrawal et al. (2024); Xue et al. (2019); Lu et al. (2021); Gao et al. (2022); Tikhonova et al. (2019); Hall et al. (2022); Coskun et al. (2014); Liu et al. (2022); Omatsu et al. (2014); Baryawno et al. (2019); Baccin et al. (2020); Zhong et al. (2020); Silberstein et al. (2016); Efremova et al. (2020); Browaeys et al. (2020); Wolock et al. (2019)
Spatial proteomics	FL and adult BM	Mouse and human	Molecular, functional and spatial information	Technology is still under development. Single-cell resolution is only achieved for selected markers. Hence, cellular identification can be difficult	Kayvanjoo et al. (2024); Bandyopadhyay et al. (2024)

Within the subaortic mesenchyme, mesodermal-derived PDGFRα^+^ cells (Chandrakanthan et al., 2022) and NG2^+^ PDGFRβ^+^ cells (overlapping with NG2^+^RUNX1^LOW^CD146^+^ cells) (Gonzalez Galofre et al., 2024; Sa da Bandeira et al., 2022) have been shown recently to support HSC specification, are proximal to the hemogenic endothelium and required for optimal specification (Clements and Khoury, 2024; Fadlullah et al., 2022). The EHT supportive ability of mesodermal-derived PDGFRα^+^ stromal cells seems to rely on PDGFRα, BMP, WNT and NOTCH signaling pathways (Chandrakanthan et al., 2022). Interestingly, mesodermal-derived PDGFRα^+^ stromal cells are present in the AGM during E10.5-E11.5, i.e., during EHT, and by E13.5 are replaced by neural crest-derived PDGFRα^+^ stromal cells (Chandrakanthan et al., 2022). In contrast to mesodermal-derived PDGFRα^+^, neural crest-derived PDGFRα^+^ stromal cells do not support EHT, as shown in reaggregate co-cultures (Chandrakanthan et al., 2022). As suggested by Chandrakanthan et al., this opens up the intriguing possibility that the replacement of mesodermal-derived with neural crest-derived PDGFRα^+^ stromal cells constitutes a regulatory mechanism selected to actively control HSC specification confining it to a very defined developmental window (Chandrakanthan et al., 2022). Interestingly, in zebrafish, the PDGFβ/PDGFRβ axis mediates HIF1α-regulated signaling axis leading to IL-6/IL-6R activation and controls embryonic HSPC production (Lim et al., 2017).

The generation of stromal clones from the surrounding aortic tissues uncovered the presence of critical factors as bone morphogenic protein-4 (BMP4) (Oostendorp et al., 2002; Oostendorp et al., 2005; Ohneda et al., 2000; Renstrom et al., 2009; Istvanffy et al., 2011), highly expressed in the aortic ventral mesenchyme of avian, mouse, zebrafish and human embryos (Pimanda et al., 2007; Marshall et al., 2000) and active in transplantable HSCs in mouse embryos at embryonic day of development (E) 11 (Crisan et al., 2015). Moreover, BMP signaling contributes to AGM polarity in zebrafish (Wilkinson et al., 2009). The use of explant cultures derived from the aorta-gonad-mesonephros region (AGM), developed by the laboratory of Alexander Medvinsky (Taoudi and Medvinsky, 2007; Taoudi et al., 2008; McGarvey et al., 2017), allowed to show that BMP4 is required in HSC specification as HSC potential was devoid in AGM explants treated with gremlin (a BMP antagonist) (Durand et al., 2007). Analysis of gene regulatory networks supported that the BMP signaling pathway and the SCL transcriptional network regulate RUNX1 activity (Pimanda et al., 2007). Importantly, the subaortic mesenchyme triggers RUNX1 expression on aortic ECs. RUNX1 marks the hemogenic endothelium and is critical for HSC specification and dispensable in later hematopoietic stages (Chen et al., 2009; North et al., 2002; Lancrin et al., 2009). Furthermore, GATA3 binds to enhancer elements in the *Runx1* locus in the HSC microenvironment, suggesting a role for RUNX1 also in the stromal cells that regulate EHT (Fitch et al., 2020).

Interestingly, BMP signaling must be downregulated following EHT to allow further HSC maturation and mediated at least in part by NOGGIN in mice (Souilhol et al., 2016a) and FGF signaling in zebrafish (Pouget et al., 2014). Additional work in avian, zebrafish and mouse embryos unveiled that the Hedgehog (Hh) signaling pathway regulated by ventral tissues is key in HSC specification (Pardanaud and Dieterlen-Lievre, 1999; Peeters et al., 2009; Wilkinson et al., 2009; Gering and Patient, 2005) while dorsal tissues exert an opposing effect, dorsoventrally polarizing the aorta (Jaffredo et al., 2013). The subaortic mesenchyme would be the source for ventral BMP4 (Marshall et al., 2000; Wilkinson et al., 2009; Durand et al., 2007), while Sonic Hedgehog (Shh) would be produced in the developing gut (Peeters et al., 2009) and both responsible for HSC specification in the ventral aspect of the dorsal aorta. Interestingly, in zebrafish embryos, the Hh pathway inhibits the hematopoietic program later during development in the dorsal aspect of the aorta, which highlights the fine-regulation of HSC specification (Wilkinson et al., 2009).

Furthermore, WNT16-dependent somite patterning and NOTCH signaling activation by DeltaC and DeltaD contributes to HSC specification (Clements et al., 2011). The intermediate mechanistic steps are still unknown (Clements and Khoury, 2024; Clements et al., 2011). The urogenital ridges in the AGM in mice also promote HSC specification although the exact involved mechanisms remain unclear (Clements and Khoury, 2024; Souilhol et al., 2016a).

Before the fusion of the paired aortas, all the aortic ECs present at that stage derive from the splanchnopleural mesoderm (Jaffredo et al., 2013). Later during development there is a replacement of the dorsal ECs with somitic mesoderm derived ECs (Pardanaud et al., 1996; Pouget et al., 2006). Particularly, in seminal experiments, Pardanaud and Dieterlen-Lievre showed in avian embryos that dorsal ECs derive from the somitic mesoderm, while ventral ECs derive from the lateral mesoderm ECs, which contributes to the dorsoventral asymmetry (Pardanaud et al., 1996). In zebrafish and mice, the EC replacement precedes HSC specification (Chandrakanthan et al., 2022; Sahai-Hernandez et al., 2023; Nguyen et al., 2014) contributing to dorsoventral polarity and influencing HSC specification.

The hemogenic endothelium (HE) undergoes an EHT to render the first HSCs defined by their ability to engraft and repopulate the hematopoietic system of a lethally irradiated recipient. The first functional HSC is detected at E11 in mouse embryos and EHT can be visualized as c-Kit^+^ clusters budding into the lumen of the aorta forming intra-aortic clusters, IACs (Medvinsky et al., 2011) (Figure 2). IACs emerge from the ventral aspect of the dorsal aorta (Medvinsky et al., 2011; Medvinsky and Dzierzak, 1996). The lack of IACs in *Runx1* ^*−/−*^ mouse embryos (Chen et al., 2009; North et al., 1999; Yokomizo et al., 2001) and the block in EHT observed in zebrafish embryos following *runx1* inhibition by morpholino (Kissa and Herbomel, 2010) demonstrated a key role of *Runx1* during EHT. NOTCH signaling is also required in EHT as shown via morpholino knockdown in zebrafish embryos (Bertrand et al., 2010). All components in the NOTCH signaling pathway including *Notch* ligands (*Jag1* and *Jag2*), *Notch1* receptor and NOTCH transcriptional targets (including *Hes1* and *Gata2*) are expressed in the ventral aspect of the aorta (Robert-Moreno et al., 2005; Robert-Moreno et al., 2008; Hadland et al., 2022). GATA2 is critical in HSC emergence and regulates *Runx1* expression (Nottingham et al., 2007), directly linking NOTCH activation with EHT. Furthermore, enforced *Gata2* and *Runx1* expression partially rescues *in vitro* the hematopoiesis defects driven by *Notch1* and *Jagged1* deficiency (Robert-Moreno et al., 2008; Nakagawa et al., 2006). Importantly, HSC specification is tightly connected with the arterial endothelial identity and suppression of NOTCH signaling via *CoupTFII* deficiency leads to the acquisition of arterial characteristics by the venous endothelium and emergence of venous hematopoietic clusters (You et al., 2005). In addition, JAG1 was shown to be required in embryonic hematopoiesis both in zebrafish and mice (Robert-Moreno et al., 2008). Similar to BMP signaling, NOTCH signaling needs to be downregulated to allow further HSC maturation (Richard et al., 2013; Souilhol et al., 2016b; Porcheri et al., 2020; Lizama et al., 2015; Ditadi et al., 2015; Kobayashi et al., 2014; Gama-Norton et al., 2015; Sacilotto et al., 2013; Thambyrajah and Bigas, 2022). Interestingly, the downregulation of NOTCH signaling may be driven by JAG1/NOTCH1 cis interaction (Ghersi et al., 2023).

During the recent years the role of sterile inflammatory signals on HSC specification have gained much attention (Hou et al., 2020; Espin-Palazon et al., 2014; Li et al., 2014; Mariani et al., 2019). Endothelial and myeloid cells (such as macrophages and primitive neutrophils) produced cytokines (including IFNα, IFNγ, NFκβ and TNFα) and affect HSC specification. Some of these cytokines (e.g., NFκβ and TNFα) converge on modulating NOTCH signaling in HSPCs in mice and zebrafish (Clements and Khoury, 2024; He et al., 2015) and are required in HSC specification. Additionally, myeloid populations contribute with matrix metalloproteases (Mmp2 and 9) that facilitate EHT and the migration of HSCs out from the dorsal aorta, as shown in zebrafish (Theodore et al., 2017). Interestingly, YS-derived macrophages, whose migration to the aorta depends on the chemokine receptor CX3CR1, constitute the largest hematopoietic cell population in the AGM and interact with IACs (Mariani et al., 2019). Notably, macrophage depletion in E10.5 AGM explant cultures blocks HSC specification *in vitro* (Mariani et al., 2019), supporting their role as part of the AGM HSC-specification niche.

Although it cannot be defined as a specific niche effect, the presence of blood circulation induces shear stress on the hemogenic endothelium, which activates the nitric oxide (NO) signaling pathway and plays an important role in EHT (Niranjan et al., 1995; Adamo et al., 2009; North et al., 2009). Blocking NO signaling pathway in *NO synthase3* deficient mice and by the administration of L-NAME *in vivo* results in the lack of IACs (Adamo et al., 2009; North et al., 2009; Murayama et al., 2006). The absence of Runx1^+^ cells in zebrafish with no blood flow can be rescued via the administration of an NO donor (S-nitroso-N-acetylpenicillamine) further demonstrating that shear stress works through NO signaling pathway (North et al., 2009; Murayama et al., 2006). Moreover, compound BF170 hydrochloride has been shown to promote HSPC induction *in vitro* in zebrafish blastomere cell cultures and from mouse embryoid bodies by activating the NO signaling pathway (Liu et al., 2024).

### Fetal liver: HSC maturation and moderate expansion

#### FL-HSC expansion vs. maturation

Following EHT, recently specified HSCs and immature HSC precursors migrate to the FL (Medvinsky et al., 2011). During this migration, Integrin-β1 (ITGB1) is critically required for FL colonization as *Itgb1* deficient HSCs and progenitors are not able to home into the FL. Interestingly, the role of ITGB1 is conserved in later developmental stages and also necessary for seeding adult hematopoietic tissues (Potocnik et al., 2000). Upon colonization, the increase in repopulating units (RUs) from ∼1-2RU in the E11.5 AGM to ∼60 RUs in the E12.5 FL (Rybtsov et al., 2014) and ∼1,000 RUs by E15.5 (Ganuza et al., 2020; Ema and Nakauchi, 2000), together with the fact that HSPCs are actively cycling in the FL (Bowie et al., 2006; Bowie et al., 2007; Morrison et al., 1995; Harrison et al., 1997; Rebel et al., 1996; Pietras et al., 2011; Bonkhofer et al., 2019) and the ability of FL-HSCs to outcompete BM-HSCs following transplantation into conditioned mouse recipients (Bowie et al., 2007; Rebel et al., 1996), led to the generally accepted view that HSCs rapidly expand in numbers in the FL based on symmetric cell division and that the FL constitutes and expansion niche (Ganuza et al., 2020; Ganuza et al., 2022b) (Figure 1). Challenging this view recent investigations, including ours, have disputed this dogma (Ganuza et al., 2020; Ganuza et al., 2022a; Patel et al., 2022; Ganuza et al., 2022b). Classical investigations only took into consideration the potential of transplanted embryonic tissues to repopulate conditioned recipients (Ganuza et al., 2020; Ganuza et al., 2022b). These studies did not investigate the actual fate of those HSCs and only measured their engraftment potential as a readout. Lineage-tracing studies employing multicolor *Confetti* allele and CRISPR/Cas9-based DNA barcoding allele to label hematopoietic progenitors during mouse ontogeny revealed that the number of hematopoietic progenitors with actual contribution into the adult HSC pool is much larger prior to EHT than previously assumed and that this pool modestly expands in the FL (Ganuza et al., 2022a; Patel et al., 2022; Ganuza et al., 2017) (Figure 1). This indicates that in the early FL (E12.5-E14.5), HSC precursors fated to contribute to the adult HSC pool are just not mature enough to repopulate the hematopoietic system of a myeloablated recipient at the moment of transplantation. But if they were to stay in the embryo they would have matured and acquired engraftment ability (Kieusseian et al., 2012). Thus, the increase in RUs observed from E12.5 to E15.5 is likely a result of the maturation of immature FL-HSC precursors rather than of a dramatic HSC symmetric expansion (Ganuza et al., 2020; Ganuza et al., 2022b). Further supporting this maturation process, employing explant-reaggregate cultures paired with limiting dilution transplantation, the Medvinsky’s laboratory reported that the number of HSC precursors (pre-HSC) in the AGM expands from E9.5 to E11.5 leading to 60 pre-HSCs at E11.5. This matches the total number of RUs in the E12.5 FL (Rybtsov et al., 2014), strongly indicating that the initial increase in RUs detected in the FL from E11.5 to E12.5 is due to HSC maturation rather than based on symmetrical HSC cell division. This further supports the role of the FL as a maturation niche (Ganuza et al., 2020; Ganuza et al., 2022b).

FL-HSCs and progenitors are actively dividing. Particularly the c-Kit^+^ HSPC population divides four times as a bulk population from E12.5 to E14.5 *in vivo* based on H2B-GFP-based tracking of cell divisions (Ganuza et al., 2022a). Yet, E14.5 FL-HSCs are biased to differentiate and to undergo asymmetric cell division *in vitro* rather than symmetrically dividing (Ganuza et al., 2022a). Thus, it is likely that *in vivo* most of these cell divisions do not result in the expansion of the FL-HSC pool with actual contribution to adult HSCs. Particularly, HSC and progenitors with contribution to the adult HSC pool only duplicates from E12.5-E15.5 as seen by multicolor-Confetti lineage tracing (Ganuza et al., 2022a). Interestingly, adult-fated HSCs minimally contribute to fetal hematopoiesis, which instead is mostly sustained by HSC-independent progenitors that establish a hierarchical hematopoietic structure in the FL (Yokomizo et al., 2022; Ulloa et al., 2021). In particular, lineage tracing in *Tg*(*drl:creERT2;ubi:lox-GFP-lox-mCherry*) reporter zebrafish indicated that definitive HSPCs negligibly participates in embryonic lymphomyelopoiesis (Ulloa et al., 2021) and depletion of HSCs via nitroreductase/metronidazole administration did not affect embryonic lymphomyelopoiesis (Ulloa et al., 2021) as previously observed in mice (Ganuza et al., 2022b; Chen et al., 2011). Likewise, lineage tracing in *Hlf* ^*creERT2*^;*ROSA26* ^*tdTomato*^ mice also showed limited contribution of HSC to FL hematopoiesis (Yokomizo et al., 2022). Additionally, transcriptional profiling of the HSC ontogeny also supports the presence of important differences among FL and adult hematopoiesis (McKinney-Freeman et al., 2012). Overall, these new body of data is important to interpret the actual role of FL-HSC niche on HSC behavior and suggests that the FL HSC-niche only supports a moderate expansion of HSCs fated to contribute to the adult-HSC pool.

#### FL-HSPC niche components and experimental approaches

**FIGURE 3:**
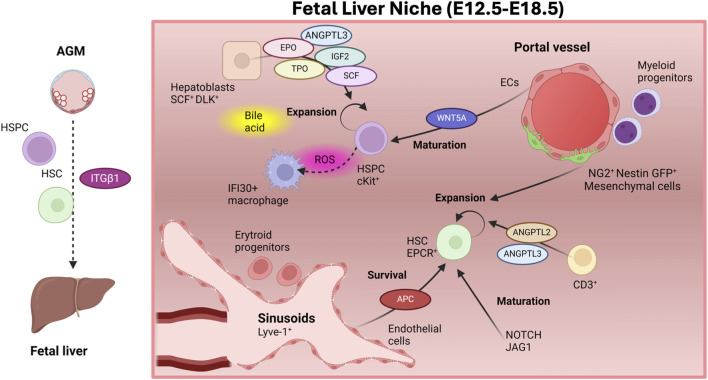
The fetal liver niche. Integrin-ẞ1 facilitates the migration of HSCs and immature HSC precursors from the AGM to the fetal liver (FL). In the FL, HSCs mature and expand in numbers, although the pool of HSCs with contribution to adult hematopoiesis moderately increases. HSCs are found close to sinusoids, where ECS sustain their survival by producing activated protein C (APC). Other FL niche components as CD3^+^ and NG2^+^ Nestin^+^ mesenchymal cells promote their expansion, while NOTCH activation is important in FL-HSC maturation. Endothelial cells in the portal vessels are involved in HSPCs maturation via activation of the WNT pathway. Many growth factors secreted by hepatocytes along with bile acid mediate the expansion of HSPCs. HSPCs are also found in close proximity to macrophages, which contribute to the scavenging of reactive oxygen species (ROS), produced by expanding HSPCs. Figure created with Biorender.

The adult liver is mainly composed of hepatocytes, cholangiocytes (biliary epithelial cells), ECs and multiple hematopoietic cell lineages (Fomin et al., 2017). In the FL, hematopoietic cells constitute the major cellular compartment, are scattered in the parenchyma and their presence strongly decrease during adulthood (Agrawal et al., 2024; Fomin et al., 2017). The human FL is also enriched in hepatoblasts (hepatocytic precursors that generate hepatocytes and cholangiocytes) (Wauthier et al., 2008). Fetal and adult hepatocytes exhibit many functional differences (Turner et al., 2007; Schmelzer et al., 2006; Schmelzer et al., 2007). FL-ECs include ECs lining lymphatic vessels, large blood vessels (i.e., portal veins and hepatic artery) and mostly sinusoidal ECs (Elvevold et al., 2008). Anatomically, FL-erythroid precursors localize close to hepatocytes and sinusoids (Orlic et al., 1982), while myeloid precursors associate to portal vascular structures (Kamps et al., 1989), and B-cell progenitors are found more dispersed in the parenchyma (Kamps and Cooper, 1982). Although mouse FL-HSPCs seem to associate with pericytes in portal vessels (Khan et al., 2016), the location of human HSPCs is incompletely understood (Fomin et al., 2017) (Figure 3).

To identify specific cellular and molecular components of the FL-HSC niche, pioneering investigations derived FL stromal cell lines and assessed their ability on supporting HSC repopulating activity following their co-culture with HSCs as an indirect measurement of their role as HSC-niche components (Gao et al., 2018). FL-derived immortalized stromal cell lines show high heterogeneity in their hematopoietic supportive ability. Some of them, such as mesenchymal and epithelial cells, sustain the repopulating activity of human and mouse HSPCs, indirectly supporting their role in the FL-HSC niche (Moore et al., 1997a; Chagraoui et al., 2003; Moore et al., 1997b; Wineman et al., 1996; Nolta et al., 2002; Hackney et al., 2002). While FL stromal cells do not trigger HSC expansion, SCF^+^ DLK^+^ FL hepatoblasts yield a ∼20-fold expansion of mouse HSPCs *in vitro* after 2–3 weeks of co-culture. Hepatoblasts express many growth factors that support HSCs including angiopoietin-like 3 (ANGPTL3), insulin-like growth factor 2 (IGF2), thrombopoietin (TPO), erythropoietin (EPO) and stem cell factor (SCF or C-KIT-ligand) (Chou and Lodish, 2010; Chou et al., 2013; Zhang and Lodish, 2004; Sugiyama et al., 2011). SCF receptor (c-KIT) is required in FL hematopoiesis as shown in *c-Kit* deficient mice (Agrawal et al., 2024; Fantin et al., 2021). Additionally, FL sinusoidal ECs promote the survival of FL-HSCs via activated protein C (APC) production, which exerts an antiapoptotic effect on EPCR^+^ HSCs inducing protease-activated receptor 1 (PAR-1) mRNA expression in HSCs(Iwasaki et al., 2010). EPCR^+^ HSCs rapidly lose *in vitro* their long-term repopulation ability unless co-cultured with FL-Lyve-1^+^ endothelial cells, further implying a role of the sinusoidal niche in FL-HSC self-renewal activity (Iwasaki et al., 2010).

Investigations in the caudal hematopoietic tissue (CHT) in zebrafish, organ equivalent to the mammalian FL and where HSCs expand, uncovered various cellular components and chemokines in HSPC proliferation and CHT colonization, including *ccl25b* (Xue et al., 2017) (homolog to *ccl2*1) and CXC chemokine ligand *cxcl8* (Blaser et al., 2017). *ccl25b* expression in CHT endothelial cells (regulated by *klf6a* transcription factor) facilitates HSPC lodgement and proliferation via *ccr7* (Xue et al., 2017). The CCL21/CCR7 interaction also allows mammalian HSPC expansion *in vitro* (Xue et al., 2017). cxcl18 and its receptor cxcr1 are both produced by CHT endothelial cells. Interestingly, cxcl18 activates cxcr1, which stimulates cxcl12 production on CHT ECs, promoting HSPC to colonize the CHT via increased “endothelial cuddling” of HSPCs (Blaser et al., 2017). In this process, ECs surround a single HSPC bound to a mesenchymal stromal cell. This orients the cell division of the HSPCs as shown by live imaging (Tamplin et al., 2015). Likewise, CXCL12 is critical in many aspects of adult HSC biology such as migration, niche retention and proliferation/quiescence (Zhang et al., 2016).

More recently, the use of algorithms aimed to detect cell interactions based on the expression of ligand-receptors among interacting cells and multiplexed fluorescence *in situ* hybridization methods further exposed the interaction among ECs and HSPCs both in the zebrafish CHT (Xue et al., 2019) and in the mouse FL (Lu et al., 2021). Molecularly, these studies highlight that integrin signaling and Smchd1 co-regulate both HSPCs and neighboring cells in the CHT (Xue et al., 2019), whose relevance will need to be evaluated. Additionally, at least one EC seems to be in direct contact with each HSC in the mouse FL (Lu et al., 2021). Among these mouse FL ECs, both arterial ECs and sinusoidal ECs were found as part of the HSC-niche (Lu et al., 2021). The role of ECs in the human FL-HSPC niche seems preserved as co-culture of human FL-ECs with FL-derived immature CD43^+^CD45^−^ HSPCs support their maturation into CD45^+^CD34^+^ HSPCs. Meanwhile, ECs derived from other tissues do not support HSPC maturation, suggesting a specific role for the FL-endothelium, which is mediated by endothelial-produced WNT5A (Choi et al., 2021). In this context, ATF4 transcription factor was shown to play a role in the FL-niche both in stromal and ECs as *Atf4* ^*−/−*^ FL stromal and ECs lack the ability to sustain HSC repopulating activity *in vitro*. This may be mediated by their inability to trigger *Angptl3* cytokine expression, a positive regulator of HSC activity (Zhao et al., 2015).

*In vitro* co-culture experiments also exposed a role for CD3^+^ hematopoietic FL cells in the HSPC-niche as they facilitate HSC expansion (Zhang et al., 2006). CD3^+^ cells produce high levels of *Angptl2* and *Angptl3* (Zhang et al., 2006). Culture of HSCs with ANGPTL2 and ANGPTL3 together with other saturating factors allows a ∼20–30-fold expansion of LT-HSCs (Zhang et al., 2006) reinforcing the role of FL-CD3^+^ hematopoietic cells in HSC expansion.

Overall, *in vitro* co-culture studies indirectly implicated multiple FL HSPC-niche components. Yet, this approach fails to provide information on actual cell-contact or proximity. Here, other methods allowing spatial analyses, such as immunohistochemistry (IHC) and immunofluorescence (IF), offer key information on niche composition. Spatially, mouse EPCR^+^ HSCs and CD150^+^CD48^−^Lin^−^ lie close to Lyve-1^+^ FL sinusoids (enriched for APC and extracellular matrix, ECM), indicating that FL-HSCs reside in a perisinusoidal FL niche (Gao et al., 2018; Iwasaki et al., 2010; Lee et al., 2021) (Figure 3). Analogously, in zebrafish, HSPCs localize with vascular ECs as shown by time-lapse imaging (Xue et al., 2017). More recently, rare perivascular NG2^+^ Nestin-GFP^+^ mesenchymal stem cells, which associate with the portal vessel in the FL, were shown to support HSCs in the FL-niche (Khan et al., 2016). These FL Nestin-GFP^+^ cells, transcriptionally similar to adult BM Nestin-GFP^+^ but more proliferative, expand in number from E12 to E14.5. *In vivo* depletion of these cells, via specific diphtheria toxin expression restricted to NG2^+^ cells in *NG2-Cre/iDTA* mice, decreases HSC numbers in E14.5 FL demonstrating a regulatory role of Nestin^+^ cells at this developmental stage (Khan et al., 2016).

As a key approach to investigate HSC niche components (Sanchez-Lanzas et al., 2022), knockout murine models perturbing the numbers of candidate niche components and deleting critical factors (e.g., SCF, critical regulator of adult HSC activity), from candidate niche cells have supported the relevance of various FL components as integral part of the FL-HSC niche. Particularly, loss of hepatoblasts in *Map2k4* null embryos reduced the levels of HSC-supportive cytokines and compromised HSPC proliferation (Sugiyama et al., 2013). Additionally, deletion of *Scf* from hepatic stellate cells and simultaneous deletion of *Scf* from ECs and hepatic stellate cells reduced or completely depleted HSCs from the FL, respectively (Lee et al., 2021), supporting their role in the FL-perisinusoidal vascular HSC niche. In contrast, conditional deletion of *Scf* from hepatocytes, hematopoietic cells, NG2^+^ cells or ECs did not perturb the number and function of HSCs (Lee et al., 2021). Reinforcing the role of FL-ECs in the FL HSPC-niche, depletion of membrane-bound SCF from the FL niche by the specific inactivation of *Ezh2* in FL-ECs results in embryonic lethality of *Ezh2* null embryos (Neo et al., 2018). Mechanistically, *Ezh2* deletion triggers MMP9 expression depleting membrane-bound SCF (Neo et al., 2018). Furthermore, in *Itgav* and *Postn*-deficient mice FL-HSCs proliferate faster yielding an enlarged pool of FL-HSCs. POSTN expression is mainly localized to the FL vascular endothelium, supporting its role in controlling the HSC pool size (Biswas et al., 2020). These data also highlight an inhibitory role of the Periostin/Integrin-αv pathway on the proliferation of FL-HSCs.

In the FL, HSPCs exhibit an increased protein synthesis rate (Agrawal et al., 2024). In this scenario, properly regulating endoplasmic reticulum (ER) stress is critical to maintain HSC function as ER stress is triggered by the accumulation of misfolded proteins (Miharada et al., 2014). In the FL, HSPCs take advantage of the presence of FL bile acids (BAs, synthesized from cholesterol) to act as chemical chaperones and inhibit protein aggregation (Sigurdsson et al., 2016). Accordingly, reduced BA levels in *Cyp27a1* ^*−/−*^ mice (compromised in their ability to synthesize cholesterol) trigger ER stress and reduces the number of FL-HSCs *in vivo*, demonstrating that BAs are critical in the expansion of the FL-HSPC population (Sigurdsson et al., 2016). These data highlight an indirect role of the FL cells implicated in BA metabolism, including hepatoblasts and hepatocytes, in HSC maintenance.

Additionally among FL hematopoietic cells, FL macrophages, which promote erythrocyte maturation in the developing FL (Saffarzadeh et al., 2020), were recently shown to interact with HSPCs in the FL via spatial proteomics coupled with single-omics (Kayvanjoo et al., 2024). In the human FL, HSPCs are also found in close proximity to IFI30^+^ macrophages (Cacialli et al., 2021) (Figure 3). Interestingly, *ifi30,* expressed by the vascular CHT niche in zebrafish, contributes to recycling reduced glutathione (GSH) (Cacialli et al., 2021). GSH is critical to scavenge reactive oxygen species (ROS) produced by HSPCs expanding in the CHT (Cacialli et al., 2021). Connexin channels (e.g., Cx41.8) in HSPCs enable this process (Cacialli et al., 2021). Additionally, CCR4 ligands CCL17 and CCL22 chemokines are highly expressed in hematopoietic cells in the E12.5 FL in mice (Konno et al., 2020). They likely play a role in the migration and retention of HSPCs in the FL as *in utero* administration of anti-CCR4 neutralizing antibody in pregnant mice compromises the number of FL-HSPCs (Konno et al., 2020). More recently, single-cell analyses combined with spatial transcriptomics identified a mouse FL-HSC niche composed of hepatoblasts, stromal cells, ECs, and macrophages supporting HSC/MPPs (Agrawal et al., 2024; Gao et al., 2022). Furthermore, many hematopoietic cells in the FL, including HSCs, produce JAG1, a major NOTCH ligand whose expression is lost in adult BM. *Jag1* deficient FL-HSCs exhibit compromised engraftment ability and downregulation of important hematopoietic factors (*e*.*g*., *Gata2*, *Mllt3*, and *HoxA7*) indicating that JAG1 mediated NOTCH activation is important in FL-HSC maturation (Shao et al., 2023). Additionally, mice lacking the *Notch1* transcriptional activation domain and *Rbpj* deficient mice show lower numbers of HSCs at E14.5 even though they exhibit no defects during specification and migration to the FL (Gerhardt et al., 2014). Thus, in the FL HSPC-niche, NOTCH signaling pathway, which it is required iteratively at different stages of developmental hematopoiesis, also plays a role in FL-HSPC expansion and/or maturation.

### Fetal spleen: limited supportive niche activity for HSCs

Synchronously to the FBM, the fetal spleen starts to receive HSCs from the FL at E15.5 as shown by repopulating activity detected at E15.5 which increases until E17.5 and remains detectable over the first couple of weeks post-birth (Christensen et al., 2004; Morita et al., 2011; Ikuta and Weissman, 1992; Wolber et al., 2002). The specific role of these HSC reservoir in the spleen is unclear but splenectomy in neonatal mouse pups does not result in a reduction of HSC numbers in the perinatal BM, indicating that the spleen is not an intermediate migration location for HSCs before moving to the BM (Bertrand et al., 2006). Furthermore, organ culture systems of fetal spleens are not capable of sustaining HSC repopulating activity following *in vitro* culture (for 4 days) while FL can (Bertrand et al., 2006). Additionally, spleen-derived stromal cell lines support myeloid differentiation but not HSC repopulating activity indirectly indicating that the fetal spleen niche is less supportive than the FL niche (Bertrand et al., 2006).

### Fetal bone marrow: colonizing the final destination

The adult BM has been widely investigated over the last two decades. A large body of studies have shown that HSCs reside mostly in perivascular niches, both arteriolar and sinusoidal, during homeostasis in the adult BM (Sanchez-Lanzas et al., 2022; Morrison and Scadden, 2014; Lucas, 2017). Adult HSC niche components include sympathetic nerves, osteoblasts, stromal cells such perivascular stromal cells and sinusoidal endothelium, and hematopoietic cells including macrophages, megakaryocytes, T-regulatory cells and neutrophils (Sanchez-Lanzas et al., 2022; Casanova-Acebes et al., 2013; Nakamura-Ishizu et al., 2015; Fujisaki et al., 2011). Among stromal cells, leptin receptor LepR^+^ reticular cells and vascular ECs produce and secrete SCF which activates the c-KIT tyrosine kinase receptor in HSCs, key in regulating the self-renewal and maintenance of HSCs (Pinho and Frenette, 2019; Crane et al., 2017; Ramasamy et al., 2016; Mendez-Ferrer et al., 2020; Batsivari et al., 2020; Chen Q. et al., 2019; Liu et al., 2021; Ding et al., 2012; Comazzetto et al., 2019). Additionally, LepR^+^ cells are a source for stromal derived factor-1 (SDF1), also known as CXCL12, a chemokine critically required for homing, trafficking and maintenance of HSCs by signaling through the G-protein coupled receptor CXCR4 in HSCs (Greenbaum et al., 2013; Sugiyama et al., 2006; Lo et al., 2009). Other relevant signals and cytokines produced in the adult perivascular BM microenvironment include NOTCH ligands, Interleukin-7 (IL7), vascular endothelial growth factor (VEGF) and Angiopoietin-1 (AGPT1) (Fang et al., 2020; Tikhonova et al., 2019; Kusumbe et al., 2016; Poulos et al., 2013; Cordeiro Gomes et al., 2016; Zhou et al., 2015).

The FBM has been much less examined. Importantly, recent studies underline the presence of substantial differences among the adult and FBM both on the cellular components and at the molecular level (Hall et al., 2022; Langen et al., 2017) which have major functional implications on the behavior of the HSPCs.

#### The early FBM: migration into an “inhibitory” environment

**FIGURE 4:**
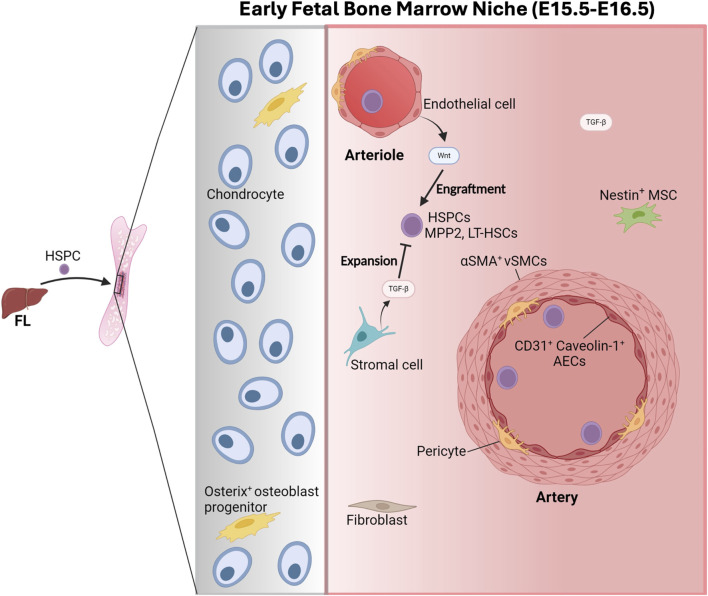
The early fetal bone marrow niche. At E15.5, the hematopoietic stem and progenitor cells (HSPCs) initiate the colonization of the recently vascularized cartilaginous femurs from the fetal liver (FL). The fetal bone marrow (FBM) is composed of chondrocytes, Osterix^+^ osteoblast progenitors, fibroblasts, Nestin^+^ mesenchymal stem cells (MSCs), pericytes and endothelial cells that support and regulate the functions of the HSPCs, mainly encompassing multipotent progenitor 2 (MPP2) and long-term hematopoietic stem cells (LT-HSCs). The secretion of WNT factors by arteriolar endothelial cells promotes the engraftment of HSPCs in the FBM. At E16.5, HSPCs are located preferentially with CD31^+^ Caveolin^+^ arterial endothelial cells (AEC), which, together with a-smooth muscle Actin^+^ vascular smooth muscle cells (aSMA^+^ VSMCs) originate arteries. The production of transforming growth factor beta (TGF-B) by stromal cells inhibits HSPC expansion. Figure created with Biorender.

The FBM stroma contains osteoprogenitors, chondrocytes, fibroblasts, pericytes and endothelial cells (Hall et al., 2022). But in contrast to the adult BM, the E16.5-P0 FBM (i.e., between E16.5 and day 0 post-birth, P0) does not seem to harbor PDGFRα^+^SCA-1^+^ mesenchymal stem cells (MSCs) and CXCL12-abundant reticular (CAR) MSCs, critical components in the adult HSC-niche which also produce ANGPT1 and SCF (Hall et al., 2022) (Figure 4).

Developmentally, coinciding with the vascularization of the femurs at E15 (Langen et al., 2017), HSPCs start to migrate from the FL and colonizing the BM around E15.5, as shown by the presence of HSC clonogenic potential (Gekas et al., 2005) and long-term multilineage repopulating activity, although at very low frequency (Christensen et al., 2004; Hall et al., 2022; Coskun et al., 2014). Arteriolar ECs facilitate the engraftment of HSPCs in the FBM by the secretion of WNT factors (Liu et al., 2022) (Figure 4). HSPCs keep translocating from the FL to the FBM until birth concomitant with a sustained decrease in the number of HSPCs in the FL from E15.5 to birth (Khan et al., 2016), while in the FBM the number of phenotypic long-term HSCs increases from ∼60 at E15.5 to ∼4,800 at P2 (Hall et al., 2022). In parallel, the number of Endomucin^+^ (EMCN^+^) vessels in the primary ossification centre of fetal femurs expands quickly from E15 (Langen et al., 2017) together with the emergence of CD31^+^ Caveolin-1^+^ arterial endothelial cells (AEC) and α-Smooth Muscle Actin+ (αSMA^+^) vascular smooth muscle cells leading to artery formation (Langen et al., 2017; Liu et al., 2022).

Intriguingly, recent investigations highlight critical differences among the HSPCs located in the FL and those in the FBM (Hall et al., 2022). The FL is mostly occupied by multipotent progenitors (MPP) type 3 (MPP3) and MPP4 until P6, when MPP3 accumulate; while MPP2s dominate in the FBM until birth when MPP3/MPP4s become prevalent (Hall et al., 2022). FBM HSPCs, including MPP2s and LT-HSCs, are vastly devoid of functional hematopoietic activity based on the lack of hematopoietic progeny following transplantation or *in vitro* clonogenic activity (Hall et al., 2022), which it is in sharp contrast with developmentally similar FL-HSPCs, suggesting the presence of “extrinsic” specific niche signals modulating HSC activity in the BM (Hall et al., 2022). Interestingly, these differences are present until birth (Hall et al., 2022). Accordingly, in contrast to FL-HSPCS, FBM HSPCs do not express stem cell programs which only become active perinatally. Moreover, the E16.5 FBM is devoid of niche cells that produce factors supportive of HSPCs (Hall et al., 2022). For instance, Nestin^+^ MSC cells present in the FBM produce lower levels of CXCL12 and other factors than adult BM stromal cells (Isern et al., 2014).

RNA-magnet based predictions (which consider the expression of known ligand-receptor pairs on interacting cells) of the HSC-niche interactome at E16.5 failed to identify significant cellular interactions, suggesting that LT-HSCs does not occupy a physically defined niche (Hall et al., 2022). However, immunostainings revealed that LT-HSCS and c-KIT^+^ HSPCs locate preferentially with Caveolin^+^ AECs at E16.5 (Liu et al., 2022), indicating a role of AECs in the colonization of the FBM.

Additionally, Osterix^+^ osteoblast progenitor cells, which are key in the adult HSC niches, are likely also important in the early FBM niche. Particularly, Osterix^+^ cells (also expressing *Pdgfrb* and *Foxc1*) are present in the E16.5 BM (Omatsu et al., 2014). Importantly, the E17.5 *Osterix* ^−/−^ FBM exhibits normal vasculature but does not harbor osteolineage cells and lacks ossification and long-term HSC repopulating activity, supporting the role of Osterix^+^ cells in the early FBM niche (Coskun et al., 2014).

Interestingly, three subclusters of LT-HSCs were identified at E16.5 including: migrating, T-cell producing, and inflamed-LT-HSCs which may comprise early thymic progenitors (ETPs) (Hall et al., 2022; Cumano et al., 2019). Although technically challenging it would be interesting to know if they occupy different specific niches in the E16.5 FBM that may determine their fate. Notably, the E16.5 FBM stromal cells are enriched for TGF-β production, with inhibitory effects on HSPC expansion (Hall et al., 2022; Blank and Karlsson, 2015). The presence of these inhibitory signals to LT-HSC and MPP2 proliferation likely hinders the function of E16.5 HSPCs (Hall et al., 2022).

#### The late FBM

**FIGURE 5:**
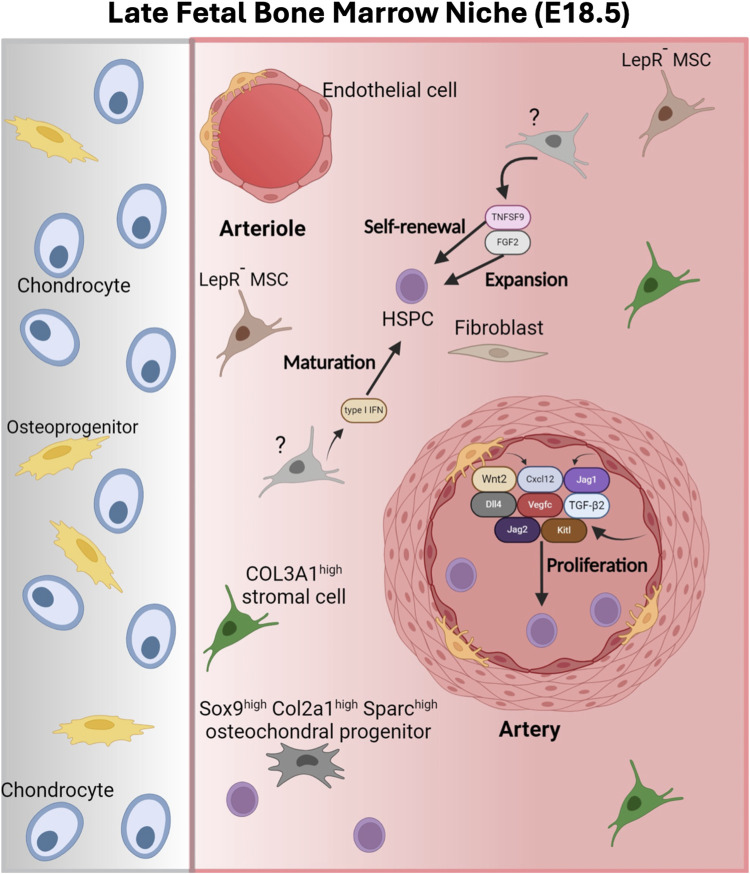
The late fetal bone marrow niche. The E18.5 FBM is characterized by the presence of Col3a1^high^ stromal cells, Sox9^high^ Col2a1^high^ Sparc^high^ osteochondral progenitors and the absence of leptin receptor^+^ (Lepr^+^) MSCs. HSPCs are found in the proximity of AECS that produce supportive BM niche factors such as C-X-C motif chemokine 12 (Cxcl12), Delta-like canonical Notch ligand 4 (DI14), Jagged canonical Notch ligand 1 and 2 (Jag1/Jag2), Kit ligand (Kit), transforming growth factor beta 2 (Tgfb2), vascular endothelial growth factor (Vegfc) and Wnt2. Additional niche factors found in the E18.5 FBM include fibroblast growth factor 2 (FGF2) and tumor necrosis factor ligand superfamily member 9 (TNFSF9), which support HSPC expansion and stem cell self-renewal, respectively. A pulse of type I interferon (IFN) promotes the maturation of HSPCs to the adult state. Figure created with Biorender.

Even though at E15.5, HSCs already started migrating to the FBM, only a small fraction of the phenotypic FBM-HSC are transplantable at E18.5 (∼%5 of HSCs in the long bones) (Medvinsky et al., 2011; Bowie et al., 2006; Mikkola and Orkin, 2006; Lessard et al., 2004; Benz et al., 2012; Qian et al., 2007). By E18.5 the FBM still harbors lower number of myeloid and megakaryocyte progenitors than the adult BM and although it contains osteolineage cell populations, it lacks LepR^+^ cells (Liu et al., 2022) (Figure 5). Osteolineage cells are initially detected perinatally (E18.5-P0) when the BM starts to transition from a cartilaginous BM (Hall et al., 2022; Hall and Miyake, 2000; Olsen et al., 2000; Matsushita et al., 2021). LepR^+^ BM cells initially locate to the metaphysis until they expand and distribute all over the adult BM (Zhou et al., 2014; Mizoguchi et al., 2014; Pineault et al., 2019; Shu et al., 2021; Matsushita et al., 2022). Various studies place quiescent adult LT-HSCs in the proximity of LepR^+^ cells in sinusoids vessels in the adult BM (Christodoulou et al., 2020) (Figure 5). Thus, the lack of these cells in the FBM likely has functional effects on LT-HSCs. As suggested by Liu and colleagues it would be interesting to unveil the molecular and cellular mechanisms that drive the migration of LT-HSCs from their arterial position in the FBM to sinusoid locations in the adult BM (Liu et al., 2022). A COL3A1^high^ BM stromal cell population (also expressing *Decorin*, *Gsn/Clec3b* and *Col1a1* and *Pdgfra* mesenchymal markers) constitutes the predominant stromal population in the E18.5 FBM (Liu et al., 2022). STAB2^high^ sinusoidal ECs, with a role in adult hematopoiesis (Tikhonova et al., 2019), were also detected in E18.5 FBM. The expression of classical niche supportive factors in E18.5 FBM is low in stromal cells and equivalent in AECs compared to the adult BM (Liu et al., 2022). At E18.5, c-KIT^+^ HSPCs still concentrate in the diaphysis of the FBM and actively proliferate in proximity to AECs (Liu et al., 2022). AECs in the E18.5 FBM produce WNT2 which can induce “β-catenin-dependent proliferation of fetal HSPCs” (Liu et al., 2022). Importantly, genetic inactivation of WNT secretion in *Wntless* knockout mice blocks HSPC expansion, as shown by reduced numbers of HSCs in E18.5 *Wntless* ^−/−^ embryos (Liu et al., 2022). E18.5 AECs also produce *Cxcl12*, *Kitl*, *Jag1*, *Jag2*, *Dll4*, *Vegfc* and *Tgfb2*, all of them classic adult BM HSC-niche factors. Overall, available data supports a role for AECs in the E18.5 FBM (Pinho and Frenette, 2019; Fang et al., 2020; Tikhonova et al., 2019; Xu et al., 2018; Guo et al., 2017). Additionally, the E18.5 FBM contains an osteochondral progenitor population (characterized by high expression of *Sox9*, *Col2a1* and *Sparc*). This population lacking expression of classical HSC maintenance factors, was predicted to interact with LT-HSCs based on RNAmagnet cell-to-cell interactions (Hall et al., 2022). By P0, both ECs and osteochondral populations were also predicted to be part of the HSC-niche (Hall et al., 2022). Interestingly these osteochondral progenitor populations and AECs produce IGF1 and IGF2, which can bind IGF1R receptors expressed by HSPCs at this developmental stage. Importantly, IGF1 is a critical regulator of adult HSC function (Hall et al., 2022; Young et al., 2021). Supporting the role of IGF1 in the perinatal HSC-niche, P0 BM-derived mesenchymal stromal cells are more efficient in supporting LT-HSCs repopulating activity *in vitro* than those derived from E16.5 and E18.5 (Hall et al., 2022). Other additional niche factors reported in the E18.5 FBM include fibroblast growth factor 2 (FGF2) and TNFSF9 which support HSPC expansion and stem cell self-renewal, respectively (Itkin et al., 2012; Kirouac et al., 2010) (Figure 5). Interestingly, a late gestational type I interferon pulse has been reported to promote the gradual transition of HSCs from fetal to adult state (Li et al., 2020). This pulse promotes the proliferation of HSPCs, enhances Major Histocompatibility I gene expression, potentially masking HSPCs from T cell-mediated destruction, and sensitizes them to FLT3^ITD^ mediated transformation (Li et al., 2020). The progressive and asynchronous nature of this transition results in cellular heterogeneity among HSPCs (Li et al., 2020).

### Facing a “new world”: changes in the bone marrow during birth

**FIGURE 6:**
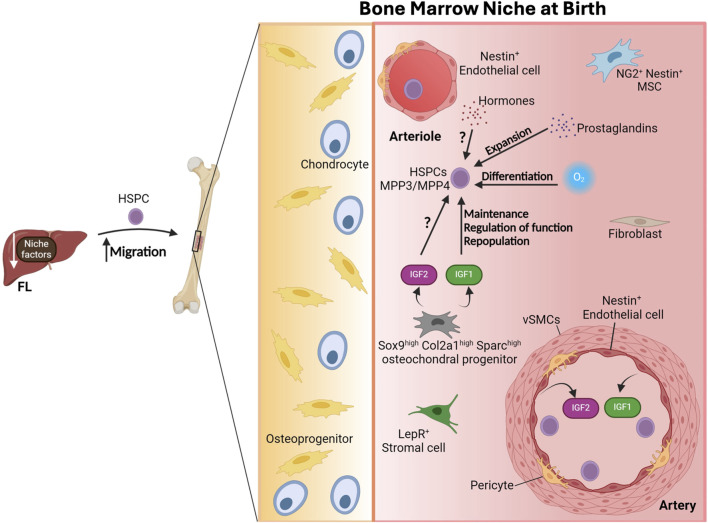
The bone marrow niche at birth. The downregulation of niche factors from the fetal liver (FL) results in the enhanced migration of hematopoietic stem and progenitor cells (HSPCs) to the bone marrow at birth. Multipotent progenitor type 3 and 4 (MPP3/MPP4) become the prevalent population of HSPCs. Neurogenin-2 (NG2^+^) Nestin^+^ mesenchymal stem cells (MSCs), leptin receptor (LepR+) stromal cells and Nestin^+^ endothelial cells are present in the natal bone marrow. Sox9^high^ Col2a1^high^ Sparc^high^ osteochondral progenitors and arterial endothelial cells (AECs) produce insulin-like growth factor 1 and 2 (IGF1 and IGF2) crucial in regulating HSPC function. Other environmental factors that impact and modulate HSPC dynamics include exposure to hormone fluctuations, prostaglandins and different oxygen levels. vSMC, vascular smooth muscle cell. Figure created with Biorender.

Around birth, the downregulation of several niche factors from FL stellate cells coincides with enhanced migration of HSCs to the BM (Lee et al., 2021). Intriguingly, there is a burst in the number of FL-HSPCs at birth, which declines by P2 in the mouse embryo (Hall et al., 2022). By P14, HSPCs are basically absent in the liver (Hall et al., 2022). Simultaneously, it is only near birth, by E19, when FBM HSPCs become fully functional and exhibit a molecular identity close to adult BM HSCs (Hall et al., 2022). This correlates with a shift in the relative frequencies and numbers of the various HSPCs and the development of a supportive BM niche, as revealed by changes in the ability of mesenchymal stromal cell derived from P0 BM to sustain LT-HSCs expansion (Hall et al., 2022), as mentioned above. However, even though the neonatal BM starts to contain niche cells expressing factors that support HSPCs, these factors are different from those present in the adult BM, emphasizing critical differences among perinatal and adult BM(185) (Figure 6). Particularly, although Cxcl12^+^ cells are present in the perinatal BM, they produce lower CXCL12 levels than the adult BM Cxcl12^+^ cells (Omatsu et al., 2014). Likewise adult-like CXCL12-abundant reticular (CAR) cells are absent from the perinatal BM (Hall et al., 2022). As aforementioned, a subtype of osteochondral progenitors supports perinatal HSPC functions by producing IGF1/2 (Hall et al., 2022) (Figure 6). It would be interesting to understand the molecular mechanisms that drive and defines these changes in the postnatal bone marrow.

It is worth highlighting that at birth mammalian newborns are exposed to major environmental changes including significant hormonal fluctuations during labor and exposure to microbes and higher oxygen levels when they abandon sterile conditions in the uterus (Hall et al., 2022). Importantly, all these factors are known to impact hematopoiesis. Particularly, ambient oxygen levels cause HSC differentiation *in vitro* (Mantel et al., 2015). Furthermore, the composition of the gut microbiota plays a role in hematopoiesis (Dominguez-Bello et al., 2010; Josefsdottir et al., 2017), thus it is likely that the changes in the microbiome of the newborn shapes the HSC-niche. Moreover, as noted by Hall and colleagues (Hall et al., 2022), prostaglandins are upregulated during birth and they have been shown to modulate HSC expansion (Ratajczak and Muglia, 2008; North et al., 2007; Porter et al., 2013). Hence, it is very conceivable that they also influence HSC biology and the BM niche at this stage.

### HSC expansion in the postnatal bone marrow and quiescence shift

As FL-HSCs migrate to the FBM, HSC numbers increase until they reach a plateau around 3–4 weeks post-birth in the mouse. Importantly, HSCs are highly proliferative during the first 3 weeks post-birth in the neonatal BM before they undergo a quiescence shift that characterizes the adult state (Bowie et al., 2006). By 4 weeks, considering that the total number of BM nucleated cells is ∼2 × 10^8^ in a mouse and that HSC frequency is 1/10,000 per nucleated BM cells (Kiel et al., 2005; Trevisan et al., 1996; Sudo et al., 2000; Sieburg et al., 2006; Szilvassy et al., 1990; Boggs et al., 1982), a mouse harbors a total of ∼20,000 HSCs. The cellular mechanisms driving this increase in the pool of HSCs are incompletely understood and relevant to regenerative medicine. The specific weight that maturation, symmetric versus asymmetric cell division, differentiation and apoptosis have in this process requires further investigation (Ganuza et al., 2020).

Lineage tracing studies tracking the global clonal complexity dynamics of hematopoietic progenitors exposed that the number of HSCs with lifelong contribution to the HSC pool expands by ∼2 fold from birth to P21 when the quiescence shift happens in mice (Ganuza et al., 2022a; Bowie et al., 2006) (Figure 1).

Similar dynamics have been detected in human based studies. Analysis of the effect of age in X-chromosome inactivation patterns in healthy women (Catlin et al., 2011) and in telomere lengths in granulocytes and lymphocytes in healthy individuals (Werner et al., 2015; Busque et al., 1996; Busque et al., 2012), showed that HSC numbers mostly expand from birth until adolescence and divide once every 40 weeks (Catlin et al., 2011) with a progressive bias towards the accumulation of HSPCs with myeloid potential with age (Werner et al., 2015). Whole genome sequencing of clones of expanded human BM-HSPCs and reconstruction of phylogenetic trees based on the analysis of the natural accumulation of somatic mutations allowed to investigate the clonal complexity origins and lineage relationships in the hematopoiesis system in humans (Lee-Six et al., 2018; Osorio et al., 2018). These studies revealed that between 50,000 and 2,00,000 clones participate in adult steady-state hematopoiesis in humans (Lee-Six et al., 2018), compatible with clonal dynamics observed in mice (Ganuza et al., 2019). Furthermore, these analyses indicated that most of the expansion in the HSC pool occurs during childhood and adolescence (Lee-Six et al., 2018). In agreement with a quiescence shift at that stage, HSC numbers would stabilize in the adulthood (Lee-Six et al., 2018). These studies also predict the presence of HSPC clones with persistent myeloid and B-lymphocyte in life, while dynamics on T-lymphoid potential were not as conclusive (Lee-Six et al., 2018; Osorio et al., 2018).

**FIGURE 7:**
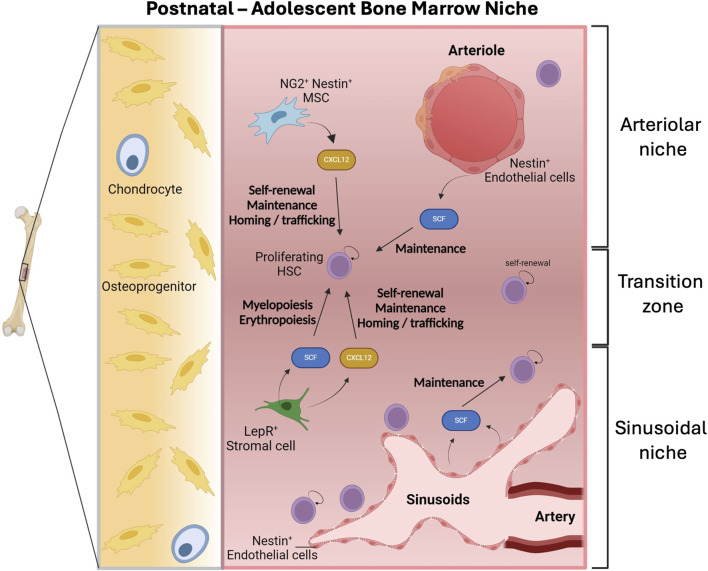
The postnatal adolescent bone marrow niche. Hematopoietic stem cells (HSCs) are highly proliferative in the neonatal bone marrow (BM) and their expansion might be associated with the development of the BM vasculature. Post-natal HSCs concentrate predominantly in sinusoidal blood vessels where Nestin^+^ endothelial cells produce stem cell factor (SCF). Additionally, the production of SCF by leptin receptor (LepR^+^) stroma cells promotes myelopoiesis and erythropoiesis. Both neurogenin-2 (NG2^+^) Nestin^+^ mesenchymal stem cells (MSC) and LepR^+^ stroma cells express C-X-C motif chemokine 12 (CXCL12) that is involved in processes such as self-renewal, maintenance, homing and trafficking of HSCs. Figure created with Biorender.

The expansion of the HSC pool may be allowed by “the development of the BM vasculature and adult niches” (Gao et al., 2018; Arai and Suda, 2007; Wilson et al., 2008) (Figure 7). In this regard, scRNA sequencing revealed that the frequencies of LepR^+^ stromal cells and ECs (arteriolar and sinusoidal) increase during the first weeks post-birth (analyzed by Kara et al. perinatally at P4, P14 and at 8 weeks post-birth) (Kara et al., 2023). These changes are accompanied by variations in the HSC-supportive ability of various populations (Kara et al., 2023). Functionally, confirming a role of Nestin^+^ cells and CXCL12 in the early postnatal BM niche, deletion of *Cxcl12* in *Nestin-CRE-ERT Cxcl12* floxed mice at P7 depletes HSCs (Isern et al., 2014), while *Cxcl12* and *Scf* deletion from Nestin^+^ cells during adulthood does not perturb HSC numbers (Ding et al., 2012; Ding and Morrison, 2013). Likewise, *Cxcl12* deletion from NG2^+^ cells at P14 depletes the HSC pool while its deletion during adulthood does not impact HSCs (Acar et al., 2015; Asada et al., 2017). Interestingly, *Scf* deletion from perinatal Nestin^+^ cells does not carry any defect (Kara et al., 2023). In the postnatal BM the patterns of expression of key adult BM factors are similar to those found in adult BM (Tikhonova et al., 2019; Baryawno et al., 2019; Baccin et al., 2020; Zhong et al., 2020; Matsushita et al., 2020). Particularly, perinatal BM LepR^+^ cells express the highest levels of *Cxcl12*, and both perinatal LepR^+^ and ECs exhibit the highest *Scf* levels, while Nestin^+^ and NG2^+^ cells do not constitute a main source of SCF (Kara et al., 2023). Interestingly, most ECs express Nestin in the perinatal BM (Mizoguchi et al., 2014; Ono et al., 2014) which becomes restricted to periarteriolar ECs and other stromal cells in the adult (Asada et al., 2017; Kunisaki et al., 2013). Notably, perinatal LepR^+^ cells-produced SCF promotes myelopoiesis and erythropoiesis but they are not required in HSC maintenance as shown by inducible conditional mouse models (Kara et al., 2023). Meanwhile, the use of *Tie2-cre*; *Scf-Ex7* ^*fl/fl*^ mice, which enables to delete the transmembrane domain of SCF from ECs (Buono et al., 2016), showed that membrane-bound SCF in ECs is required to maintain the HSC pool in the perinatal BM (Kara et al., 2023). Still, this does not exclude that soluble SCF-produced by ECs plays also a role (Kara et al., 2023). Among adult BM-ECs, SCF is mostly expressed by arteriolar ECs (Tikhonova et al., 2019; Liu et al., 2022; Xu et al., 2018; Baryawno et al., 2019), whereas at P4, sinusoidal ECs also produce it (Kara et al., 2023). Confocal imaging of *α-Catulin* ^*GFP/+*^ mice showed that, like in the adult BM, HSCs concentrate mostly in sinusoidal blood vessels in the perinatal BM (P4 and P14) (Kara et al., 2023). A small fraction of HSCs also locates to transition zone vessels, which exhibit “intermediate properties between sinusoids and arterioles” (Kara et al., 2023) (Figure 7). This may be reminiscent of the ongoing migration of HSCs from their arterial location in the FBM to sinusoids during adulthood (Liu et al., 2022).

Molecularly, important metabolic, transcriptional and epigenetic differences have been reported among FL-HSC an adult-BM HSCs which are likely linked to their proliferative stage and transition into quiescence (Manesia et al., 2015; Jassinskaja et al., 2017; Roy et al., 2021; Manesia et al., 2017). FL-HSCs contain more mitochondria, express higher levels of genes implicated in oxidative phosphorylation (OxPhos) and citric acid cycle (TCA), consume higher oxygen and produce increased ROS levels than BM HSCs (Agrawal et al., 2024; Manesia et al., 2015). Interestingly, adult HSCs display a larger collection of proteins involved in the protection against ROS-induced protein oxidation that help to preserve HSC stemness in the long-term (Jassinskaja et al., 2017). Inflammatory signaling is also upregulated in adult HSCs (Roy et al., 2021). Remarkably, *Sox17* is expressed in fetal and perinatal HSCs until ∼4 weeks post-birth concomitant with the quiescence shift, and conditional knockout of *Sox17* leads to the loss of fetal HSCs but does not impact adult HSCs when deleted later in development. This underscores the critical role of Sox17 in fetal HSCs (Kim et al., 2007).

Epigenomic analyses revealed that even though chromosomal compartments and topologically associating domains (TADs) are broadly conserved between fetal and adult HSCs, there is still an increased compartmentalization in the chromosomal compartments and more dynamic chromatin interactions within TADs in adult HSCs (Chen C. et al., 2019). This affects thousands of interactions among gene promoters and enhancers. These changes are driven by particular transcription factors, including TCF3 and MAFB in fetal HSCs and NR4A1 and GATA3 in adult HSCs (Chen C. et al., 2019).

Overall, these investigations emphasized major molecular and functional differences among fetal and adult HSCs which are highly determined and regulated by developmentally distinct HSPC niches.

## Discussion and future perspectives

From their emergence in the major embryonic arteries, HSCs and their precursors travel over multiple locations that initially allow their specification and later shape their maturation and expansion. Those HSC microenvironments provide a variety of signals that enable the fine-tuning of HSC development by temporally and spatially confining specific developmental stages, for instance by activating BMP and NOTCH signaling before EHT and blocking it afterwards to allow HSC maturation (Souilhol et al., 2016a; Richard et al., 2013; Pouget et al., 2014; Souilhol et al., 2016b; Porcheri et al., 2020; Lizama et al., 2015; Ditadi et al., 2015; Kobayashi et al., 2014; Gama-Norton et al., 2015; Sacilotto et al., 2013). Interestingly, similar pathways are iteratively employed at different developmental stages. NOTCH signaling, required during HSC specification in the AGM and HSC expansion in the FL, constitutes a clear example (Clements and Khoury, 2024; He et al., 2015; Gerhardt et al., 2014). Importantly, during the migration of HSCs and progenitors between embryonic/fetal locations, the presence of adequate niche space to allocate migrating HSCs can constitute a developmental bottleneck shaping the ability of migrating cells to realize their cellular potential and a factor to be taken into consideration (Ganuza et al., 2020).

Over the recent years, the advent of novel non-invasive lineage tracing approaches coupled with single-cell technologies are dramatically changing our view of hematopoiesis and exposing unexpected findings (Sanchez-Lanzas et al., 2022), some of which await functional validation. These techniques have revealed the presence of an unexpected degree of cellular heterogeneity at multiple developmental stages of hematopoiesis, including the detection of both transient and persistent embryonic hematopoietic populations with an HSC-dependent or independent origin (Patel et al., 2022; Yokomizo et al., 2022; Ulloa et al., 2021; Beaudin et al., 2016). This suggests the possibility of specific developmental niches facilitating their emergence and supporting these different hematopoietic populations and/or the presence of cell-intrinsic differences among precursors that make them to behave differently under the same niche inputs.

Among others, recent lineage-tracing studies provocatively indicated the presence of an HSC-independent population of embryonic multipotent progenitors (eMPPs) which was suggested to predominantly contribute to hematopoiesis in young mice with a lymphoid bias, and which also contributes to lifelong hematopoiesis (Patel et al., 2022).

Additionally, lineage-tracing analyses, next generation sequencing of naturally occurring mutations, telomere lengths and X-chromosome inactivation patterns revealed unexpected clonal dynamics on the expansion of the HSC pool with age, which affects the way we used to understand the FL as an expansion niche and rather support a major role of the FL as a maturation niche (Ganuza et al., 2022a; Catlin et al., 2011; Werner et al., 2015; Lee-Six et al., 2018). This calls for caution on focusing on the FL in a search for signals driving HSC symmetric expansion. Likewise, the lack of contribution of adult-fated HSCs to fetal hematopoiesis (Ulloa et al., 2021) and the presence of a hierarchical hematopoietic structure independent from adult-fated HSCs (Yokomizo et al., 2022) further complicates the overall picture of hematopoiesis during fetal stages, suggesting the presence of specific FL niches for each of those populations and/or cell-intrinsic differences among HSCs and other HSPCs as aforementioned.

Furthermore, lineage-tracing studies also unveiled additional layers of complexity in developmental immunity with an embryonic Flk2^+^ transient HSC population (Beaudin et al., 2016). This population exhibits multilineage potential and contributes mostly to B1a B-cells and γδ T-lymphocytes (Beaudin et al., 2016). This Flk2^+^ transient HSC population is not detected normally in the adulthood. However, the induction of inflammation *in utero* increases its persistence in the adult HSC compartment and affects postnatal immunity (Lopez et al., 2022). The effect of inflammation and infection on adult hematopoiesis has been well-established (Johnson et al., 2020) but their impact on fetal hematopoiesis and their long-term consequences on postnatal immunity is only now starting to be explored (Apostol et al., 2020; Tseng and Beaudin, 2023). In the same line, future investigations on the effect of extrinsic and environmental factors such as the establishment of gut microbiota during birth (Fernandez Sanchez et al., 2024), congenital infections (Lopez et al., 2022), hormonal changes during delivery (Calvanese et al., 2014) and inflammation, in developmental hematopoiesis, including changes in the HSC niche composition, will surely uncover important biological insights.

Importantly, over the recent years our understanding on the molecular regulation that the various HSC niches exert on HSC emergence and maturation has rendered several breakthroughs in regenerative medicine related to HSC expansion and HSC generation from other cellular sources (i.e., induced pluripotent stem cells, iPSCs) (Ng et al., 2024). HSCs have historically proved very difficult to expand and maintain in culture. Unfortunately, this implies the continuous need to search for compatible bone marrow donors to cope with the thousands of patients in dramatic need of this life saving therapy every year. Intense research on culture conditions supportive of HSC expansion recently led to protocols that significantly improve HSC expansion while maintaining their engraftment ability over long periods of time (Wilkinson et al., 2019; Sakurai et al., 2023). In parallel, several approaches have been taken to derive HSCs from other cell types. For instance, enforced expression of a cocktail of transcriptional factors supportive of HSC specification combined with their co-culture with engineered niche cells allowed to derive HSC-like cells from human iPSCs and mouse endothelial cells (Hadland et al., 2022; Sugimura et al., 2017; Lis et al., 2017; Hadland et al., 2015). Additionally, various stepwise differentiation protocols based on the subsequent and timely administration of specific developmental cues (including BMP4, ActivinA, bFGF, VEGF…) have been developed to *in vitro* mimic the transition from pluripotent stem cells to embryoid bodies, mesoderm and hemogenic endothelium and have rendered hematopoietic products close to adult mature HSCs, but with limited lymphoid production (Ditadi et al., 2015; Ditadi et al., 2017; Sturgeon et al., 2014). Very recently, a stepwise protocol has been optimized to produce long-term engrafting multilineage HSCs from human iPSCs (Ng et al., 2024). Although this work awaits to be replicated by others and to be evaluated in clinical trials, it holds the promise to provide unlimited numbers of clinically applicable HSCs for autologous transplantation avoiding serious problems related with graft-versus-host disease.

Our knowledge on the HSC niches and how they change during leukemogenesis is also being exploited to develop novel anti-leukemia therapies. Particularly, the role of the BM niche in adult leukemia is a matter of intense research, and a large body of data has exposed a critical role of the BM microenvironment in leukemia emergence, progression and development of chemoresistance. For instance, this includes how acute myeloid leukemia (AML) cells dramatically modify and highjack the BM niche to survive (Lane et al., 2009; Schepers et al., 2015; Duarte et al., 2018; Krevvata et al., 2014; Hanoun et al., 2014; Passaro et al., 2021) and the way the adipocyte niche changes following therapy promoting quiescence and chemoresistance of adult acute lymphoblastic leukemia (ALL) cells (Dander et al., 2021; Heydt et al., 2021). Likewise, in chronic myeloid leukemia (CML), CXCL12 was shown to maintain the quiescence of tyrosine kinase inhibitor (TKI)-resistant leukemia stem cells. Remarkably CXCL12 depletion from MSCs improves TKI efficacy in this context (Agarwal et al., 2019; Zhang et al., 2012; Kvasnicka and Thiele, 2004).

In contrast to adult leukemia, the specific role of cellular components supporting the transformation of HSCs and hematopoietic progenitors into preleukemic clones during embryogenesis and fetal development is widely unknown. Childhood leukemia encompass a heterogenous group of diseases. The evolution of preleukemic clones into the same type of leukemia in both monozygotic twins sharing clonal markers robustly supports a fetal origin (Greaves et al., 2003). The presence of fusion genes typically detected in childhood leukemia (e.g., ETV6-RUNX1) in mesenchymal stem cells of leukemia infant patients indicates that these mutations are acquired before HSCs emerge (Shalapour et al., 2010). The way how these mutations affect HSC development, their interactions with the FL- and BM-niches and childhood leukemia development is vastly unknown. Future investigations will surely address these critical questions in leukemogenesis. For instance, the initial steps leading to B-cell acute lymphoblastic leukemia (B-ALL) development are usually unnoticed in children. It has been recently shown that “immune stress suppresses innate immune signaling in preleukemic precursor B-cells” leading to leukemia in predisposed mice harboring pre-malignant *Pax5* ^+/−^ B-cell precursors (Isidro-Hernandez et al., 2023). Thus, understanding the molecular changes that the hematopoietic niches undergo during the earliest stages of childhood leukemia will offer new therapeutic intervention options in children.

Overall, the use of novel technologies has uncovered new insights in developmental hematopoiesis and highlighted the importance of reevaluating commonly accepted dogmas to reassign specific properties to particular developmental stages and niches. The implementation of new research tools combined with their functional validation will keep shaping our understanding on the various HSC niches. The multiple sequential anatomic locations that form the HSC niche during development provides a paradigmatic example on the highly complex and exquisitely regulated processes required to yield a functional tissue and maintain homeostasis. Furthermore, recent breakthroughs in regenerative medicine highlight the critical importance on understanding embryonic and fetal developmental processes to implement and improve life-saving cellular therapies and its potential to provide alternative anti-leukemia strategies.

## Abbreviations

AGM, aorta-gonad-mesonephros; AECs, aortic endothelial cells; BA, bile acid; BM, bone marrow; E11, embryonic day of development 11; CHT, caudal hematopoietic tissue; CAR, CXCL12-abundant reticular cells; ER, endoplasmic reticulum; ECs, endothelial cells; EHT, endothelial to hematopoietic transition; EMPs, erythroid-myeloid progenitor cells; FBM, fetal bone marrow; FL, fetal liver; HSCs, hematopoietic stem cells; HSPCs, hematopoietic stem and progenitor cells; HE, hemogenic endothelium; IHC, immunohistochemistry; IF, immunofluorescence; IACs, intra-aortic clusters; RUs, repopulating units; MSCs, mesenchymal stem cells; YS, yolk sac.
